# Individuals who see the good in the bad engage distinctive default network coordination during post-encoding rest

**DOI:** 10.1073/pnas.2306295121

**Published:** 2023-12-27

**Authors:** Siddhant Iyer, Eleanor Collier, Timothy W. Broom, Emily S. Finn, Meghan L. Meyer

**Affiliations:** ^a^Department of Psychology, Columbia University, New York, NY 10027; ^b^Department of Psychology, University of California, Riverside, CA 92521; ^c^Department of Psychological and Brain Sciences, Dartmouth College, Hanover, NH 03755

**Keywords:** default mode network, memory, resting state, affect, fMRI

## Abstract

Focusing on the upside of negative events often promotes resilience. Yet, the underlying mechanisms that allow some people to spontaneously see the good in the bad remain unclear. The broaden-and-build theory of positive emotion has long suggested that positive affect, including positivity in the face of negative events, is linked to idiosyncratic thought patterns (i.e., atypical cognitive responses). Yet, evidence in support of this view has been limited, in part, due to difficulty in measuring idiosyncratic cognitive processes as they unfold. To overcome this barrier, we applied Inter-Subject Representational Similarity Analysis to test whether and how idiosyncratic neural responding supports positive reactions to negative experience. We found that idiosyncratic functional connectivity patterns in the brain’s default network while resting after a negative experience predicts more positive descriptions of the event. This effect persisted when controlling for connectivity 1) before and during the negative experience, 2) before, during, and after a neutral experience, and 3) between other relevant brain regions (i.e., the limbic system). The relationship between idiosyncratic default network responding and positive affect was largely driven by functional connectivity patterns between the ventromedial prefrontal cortex and the rest of the default network and occurred relatively quickly during rest. We identified post-encoding rest as a key moment and the default network as a key brain system in which idiosyncratic responses correspond with seeing the good in the bad.

## Significance

Negative experiences are unavoidable, which is why effective coping strategies are key to well-being. One health-protective strategy is seeing the positive features that arise from life’s challenges. Yet, to date, the underlying mechanisms that allow some people to spontaneously see the good in the bad remain speculative. We found responses by the brain’s default network immediately after hearing about life’s hardships explain why some of us react with optimism while others with despair. Specifically, homogenous default network responses corresponded with negative reactions, whereas idiosyncratic default network responses corresponded with positive reactions. People who can see negative situations through rose-colored glasses may be able to do so, in part, by wearing their own, unique lens after the event.

It is often helpful to see the good in the bad. Emphasizing the positive consequences of a medical condition, such as how it brings into focus what matters in life, promotes mental and physical health in patients and their caretakers (1, 2). Highlighting the positive ramifications of an interpersonal betrayal fosters forgiveness and reconciliation (3, 4). Even outside of our immediate social sphere, adopting a hopeful perspective on strangers’ plights increases costly donations to relevant charities (5). While multiple pieces of evidence point to the upside of positivity in response to negative situations, the underlying neurocognitive mechanisms by which people see the good in the bad remain vastly underspecified.

To help fill this knowledge gap, we attempt to answer the following questions: How, in terms of an underlying cognitive mechanism, are some people able to see the positives of a negative experience? Where in the brain does this mechanism occur? And when during a negative experience does the mechanism come online to generate positivity? Insight into the how question may come from the broaden-and-build theory of positive emotion. This account proposes that while negative affect corresponds with prototypical thought patterns, positive affect is linked to idiosyncratic thought patterns (6). For example, normative responses to creative problem-solving are associated with negative affect (7), whereas unusual and diverse responses—relative to group norms—are tied to positive affect (8). Research on foraging behavior tells a similar story about idiosyncrasy and positivity. In both human and non-human animals, exploring atypical paths is associated with positive affect, whereas exploiting known outcomes is associated with negative affect (9–11). Collectively, work on explore-exploit behavior and the broaden-and-build theory of positive emotion suggests that people who can see the good in the bad may do so through idiosyncratic cognitive processing.

To date, this possibility has not been tested, in part, because it is difficult to measure idiosyncratic cognition “in vivo.” To overcome this barrier, we capitalized on a recent advance in computational neuroscience that is designed to detect idiosyncratic (as well as normative) cognitive processing as it naturally unfolds. Inter-Subject Representational Similarity Analysis (IS-RSA) quantifies the similarity in subjects’ neural responses as a function of a behavioral metric, such as their interpretations to stimuli (12). Here, we specifically employed an IS-RSA “Anna Karenina” model (12), so named after the opening line of Tolstoy’s famous novel, which goes, “All happy families are alike; each unhappy family is unhappy in its own way.” Although Tolstoy’s line posits greater similarity for positive vs. negative experiences, this need not be the case; the Anna Karenina model simply tests whether there is idiosyncrasy in one set of responses compared to another. For the present study’s hypotheses, our Anna Karenina model predicted that subjects who see a negative event more positively will show idiosyncratic neural responses, while subjects with more negative views will show highly similar neural responses.

If idiosyncratic cognition underlies positivity in the face of negative events, the next key question to answer is where in the brain does the idiosyncrasy occur? Idiosyncrasy may occur in the brain’s default network, an interconnected set of cortical regions associated with subjective interpretations (13), including subjective, affective interpretations (14, 15). While affective reactions (e.g., high arousal) are linked to limbic regions outside of the default network, the subjective representation of affective reactions (e.g., the construal of a high arousal state as tense vs. excited) is associated with default network regions (14–16). Moreover, individuals with similar subjective beliefs (e.g., political partisans) demonstrate neural synchrony in default network regions while processing belief-relevant stimuli (17–21). Two distinct findings, when considered together, further suggest that idiosyncratic responses in a particular portion of the default network—the ventromedial prefrontal cortex (VMPFC)—may be key to generating positivity in response to negative events. First, VMPFC increases engagement within subjects when they are explicitly instructed to try to find positive meaning (vs. control conditions) in responses to negative stimuli with high internal validity (e.g., photographs of very upsetting images (22)). Second, the VMPFC responds idiosyncratically across subjects while they view naturalistic stimuli designed to more closely mirror situations witnessed in everyday life (23). Collectively, these findings suggest idiosyncratic responding by the default network generally, and the VMPFC, particularly, might underlie seeing the good in the bad spontaneously (i.e., without instruction). To investigate these possibilities, we tested whether idiosyncratic functional connectivity (i.e., timecourse correlations reflecting coactivation) 1) between all default network regions and 2) specifically between the VMPFC and other default network regions corresponds with positive reactions to negative information.

With respect to the when question, there are two competing possibilities regarding when during a negative experience idiosyncratic default network connectivity comes online to generate positivity. One possibility is that the phenomenon occurs during encoding—in the moment of witnessing negative information. Consistent with this possibility, past work shows negative affect increases default network similarity across subjects while encoding emotional narratives (24). Greater default network similarity while listening to an ambiguous story also corresponds with more negative interpretations of it (17). Critically, however, this past work narrowly focuses on negative affect and has not investigated the key tenet of the broaden-and-build theory, which is that idiosyncratic cognitive responses should relate to positive affect.

Moreover, encoding may not be the moment in which idiosyncrasy supports seeing the good in the bad. One prediction from the broaden-and-build-theory is the “undoing hypothesis” which suggests idiosyncratic cognitive processing occurs directly after a negative experience to generate a positive interpretation (6). Other work also finds that positive interpretations can occur after a negative event to make the memory less threatening (25). Thus, a second possibility is that idiosyncratic default network connectivity spontaneously occurs directly after a negative experience to generate a more positive interpretation. This possibility is further supported by research on memory consolidation, which suggests offline processes during rest after an experience, including offline processes in the default network, shape how it is remembered (26–29). Idiosyncratic cognitive processes may therefore emerge in the default network during rest after a negative experience to help generate a more positive lens on the event. If this were the case, it would suggest the need for a paradigm shift with respect to how seeing the good in the bad is experimentally measured and manipulated: Rather than assessing the phenomenon during a negative experience, as is frequently done (17, 22, 24), researchers may want to consider assessing it post-encoding.

To test whether, where, and when idiosyncratic neural responding corresponds with seeing the good in the bad, we had subjects undergo functional MRI (fMRI) while they watched videos of patients diagnosed with cystic fibrosis discussing their experience with the condition. Using videos of cystic fibrosis patients allowed us to test our hypotheses in a context where individual differences in positive vs. negative affect meaningfully predict well-being (30, 31). That is, when caregivers stay positive, it is better for the patients’ mental and physical health outcomes as well as their own (1–3, 31–36). Here, our experimental setup mimics the type of situation a caregiver may face, in which a patient discloses difficult information to them, and the listener could (or could not) spontaneously focus on the good in the bad. If we observe idiosyncratic responding predicts positivity, it would shed light on the basic neurocognitive mechanisms that facilitate this resilient strategy.

Subjects also watched, inside the scanner, videos describing the biology of cystic fibrosis (e.g., the genetic basis of cystic fibrosis). These videos covered cystic fibrosis content that is less open to subjective interpretation than the patient videos, which allowed us to examine whether idiosyncrasy-related positivity occurs most strongly in response to negative stimuli open to interpretation. Before and after watching the videos, subjects completed rest scans. After completing the rest and video scans, subjects stepped out of the scanner and wrote descriptions of what they remembered from each video, separately. Subjects’ descriptions were submitted to a sentiment analysis that quantified negative and positive content. We were therefore able to test 1) whether idiosyncratic neural responses predict more positive reactions to patients’ experiences, 2) whether the idiosyncratic responses are present in the default network, and 3) when—during encoding or post-encoding rest—the phenomenon occurs.

## Results

### Capturing Variability in Affect.

**Fig. 1.**
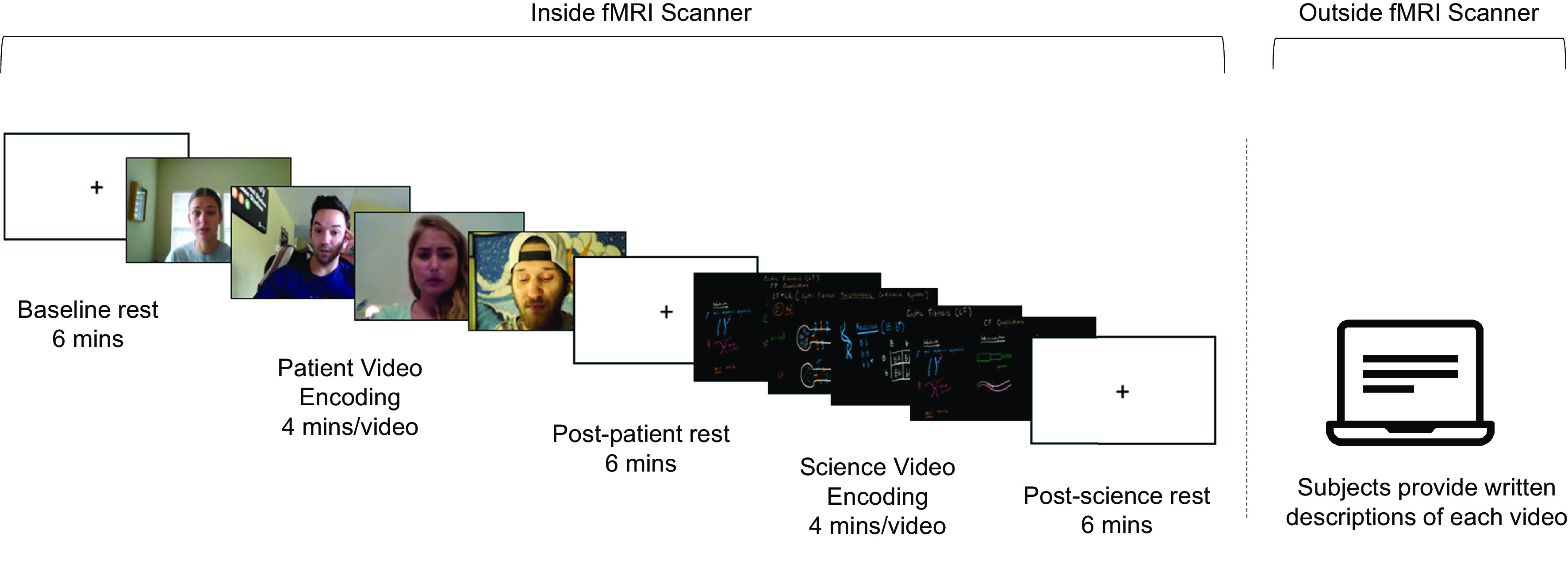
Paradigm. Patient and science encoding were interleaved with rest scans to assess offline processing. Whether subjects first completed patient or science encoding was counterbalanced across subjects. After fMRI scanning, subjects wrote down everything they remembered from each video.

To test our predictions about whether, where in the brain, and when idiosyncratic cognitive processing may generate positivity in response to negative information, we had subjects complete fMRI while undergoing the following experimental phases: The “patient encoding” phase comprised four, approximately 4-min videos of cystic fibrosis patients discussing their experience with the disease, and the “science encoding” phase comprised four, approximately 4-min Khan Academy videos describing the biology of cystic fibrosis. The order of patient and science encoding was counterbalanced across subjects, and the order of the videos within each block was randomized across subjects. Prior to these phases, subjects completed a 6-min baseline rest scan. Subjects also completed 6-min rest scans following each encoding phase, here termed “post-patient rest” and “post-science rest.” After their scan session, subjects responded to a surprise free recall prompt on a computer, in which they viewed a snapshot of each video and were asked to type everything they recalled from the video. The paradigm is depicted in Fig. 1.

To derive an objective measure of the affect in subjects’ descriptions, their written responses were submitted to a sentiment analysis, which is a natural language processing approach used to determine the degree to which language is positive, negative, or neutral. Specifically, we used Valence Aware Dictionary and sEntiment Reasoning [VADER (37)], and specifically its Sentiment Intensity Analyzer module, which was created by leveraging and improving upon lexicons and techniques from existing natural language processing models (e.g., LIWC and ANEW), independent Amazon Mechanical Turk word ratings, and machine learning text classifiers.

**Fig. 2.**
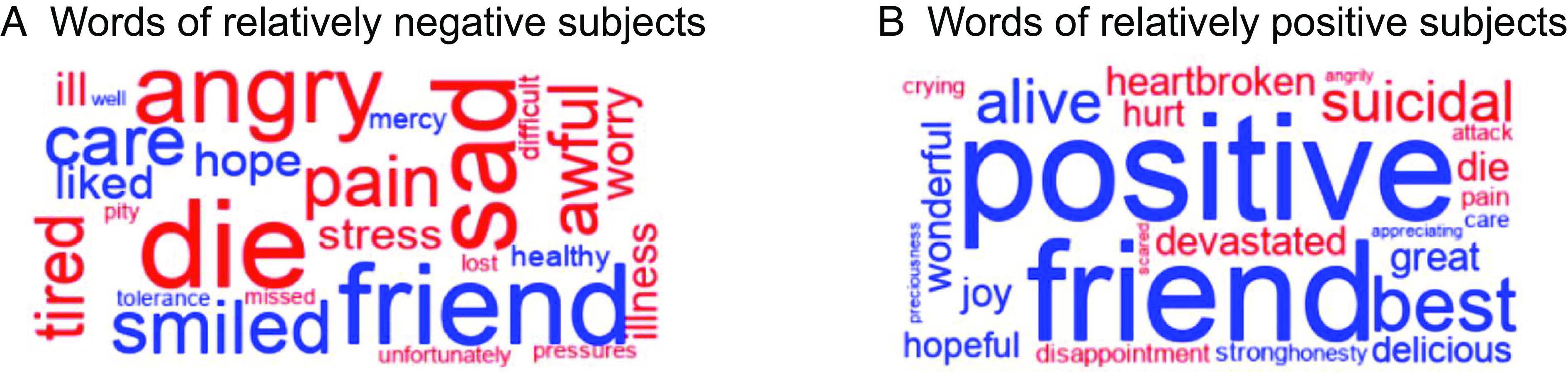
Word clouds showing words used by subjects when describing the patient videos. (*A*) Words used by subjects with the most negative descriptions. (*B*) Words used by subjects with the most positive descriptions. Negative words are shown in red; positive words are shown in blue. Font size indicates strength of affect (highly negative or highly positive).

Fig. 2 depicts the words used by two subjects to describe the patient videos, one of whom had a highly negative affect score (Fig. 2*A*) and the other a highly positive affect score (Fig. 2*B*). Negative words are shown in red, positive words are shown in blue; bigger words have stronger affect (highly negative or highly positive). It is noteworthy that subjects’ affect scores were not significantly correlated with the length of their recall (*r* = 0.243, *P* = 0.131), suggesting affect was not confounded with the amount of information recalled. Subjects’ affect scores were also not significantly correlated with the number of affective words written (*r* = 0.096, *P* = 0.554), indicating that their affect scores represent the “degree” of valence, rather than just the presence or absence of affective words. In keeping with the main analyses, all the above tests were computed using Spearman correlation, such that inferences could be drawn regarding the rank order of subjects without assuming linearity between their affect scores and connectivity idiosyncrasies.

Importantly, there was no significant correlation (*r* = −0.101, *P* = 0.536) or difference (t = −1.720, *P* = 0.093) in the affect scores between patient (i.e., patient; Mean_social_ = −0.148, SD = 1.894) and science (i.e., Khan Academy; Mean_nonsocial_ = 0.546, SD = 1.473) responses. These results suggest, respectively, that subjects’ affect in their patient descriptions is not conflated with their affect in their science descriptions and that the overall affective content in descriptions was “matched” for the patient and science videos.

### Collapsing across Individual Differences, Subjects Show Similar Functional Connectivity during Each Phase of the Experiment.

**Fig. 3.**
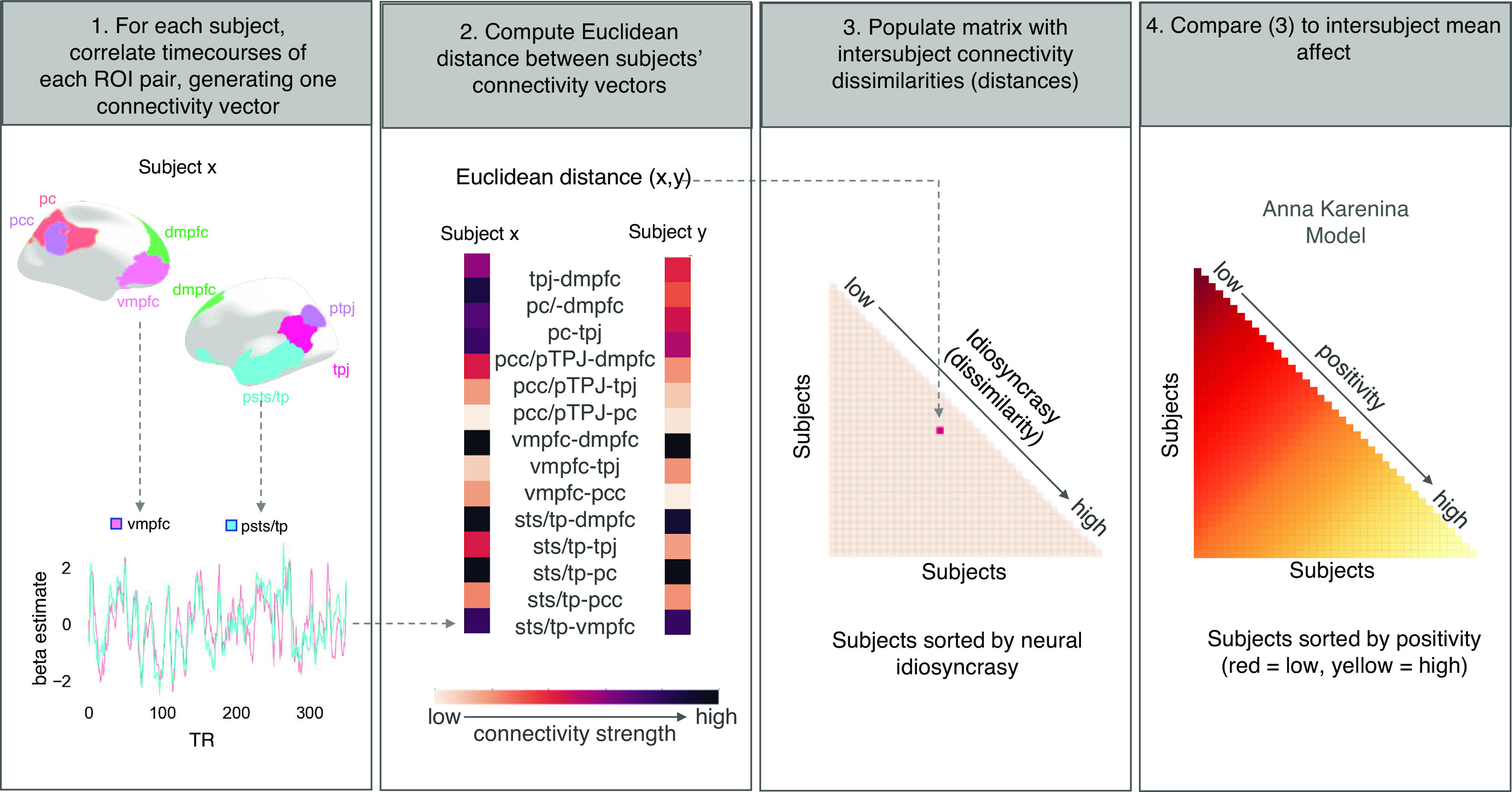
Data analytic approach involving four steps. First, for each subject and for each phase of the experiment, neural activation timeseries from the default network ROIs were extracted and correlated with one another to generate connectivity vectors. Second, the Euclidean distance metric was used to compute dissimilarity in connectivity vectors between subjects. Third, each subject pair’s connectivity dissimilarity was populated into a subject-by-subject matrix. Fourth, the inter-subject connectivity dissimilarity matrix was statistically compared to the theoretical Anna Karenina model. To examine the relative specificity of results to the default network, follow-up analyses were also conducted with limbic region ROIs.

Before testing for idiosyncrasy-related positive affect, we simply assessed the extent to which subjects exhibited similar functional connectivity profiles in default network regions, as well as limbic regions, during encoding and rest. As depicted in Fig. 3, this analysis requires first extracting, for each subject, the timecourse of neural activation [i.e., blood-oxygenation-level-depend (BOLD) signal] from each region of interest (ROI) and correlating these timecourses for each ROI-pair. Next, a vector is created for each subject, in which each vector cell is populated by the timecourse correlation of an ROI pair. All subjects’ vectors (i.e., network connectivity profiles) are correlated with one another and the median correlation, which is the summary statistic for overall inter-subject similarity, is then tested against a bootstrapped null distribution for significance. This analysis assesses the extent to which subjects’ functional connectivity profiles are similar to one another and helps ensure that across subjects, our measure of functional connectivity is reliable before examining its relationship to individual differences in affect.

Functional connectivity similarity during patient and science encoding was significant between default network regions, as well as between limbic regions [with and without nucleus accumbens (NAc); *r*’s > 0.574, *P*’s < 0.001]. Similarity in functional connectivity was also significant in these regions during each of the rest scans (*r*’s > 0.475, *P*’s < 0.001). The results so far suggest that when collapsing across individual differences, subjects show similar functional connectivity profiles to one another in brain regions relevant to subjective interpretation (default network regions) as well as those relevant to emotional reactivity (limbic regions).

### Default Network Idiosyncrasy during Post-Patient Rest Predicts Positive Descriptions of Patients’ Experiences: Inter-Subject Representational Similarity Analysis (IS-RSA).

We used an Anna Karenina model to test whether, where in the brain, and when idiosyncratic responding may predict seeing the good in the bad. Our operationalization of the model predicts that subjects with highly negative descriptions would show similar neural responding while subjects with highly positive descriptions would show idiosyncratic neural responding. The approach involves comparing the mean affect of each subject pair (“inter-subject mean affect”) with their connectivity dissimilarity (“inter-subject connectivity dissimilarity”). Note that we here use a dissimilarity measure instead of similarity, only for ease of interpretation (i.e., to reflect idiosyncrasy). Fig. 3 provides a conceptual depiction of the IS-RSA approach.

**Fig. 4.**
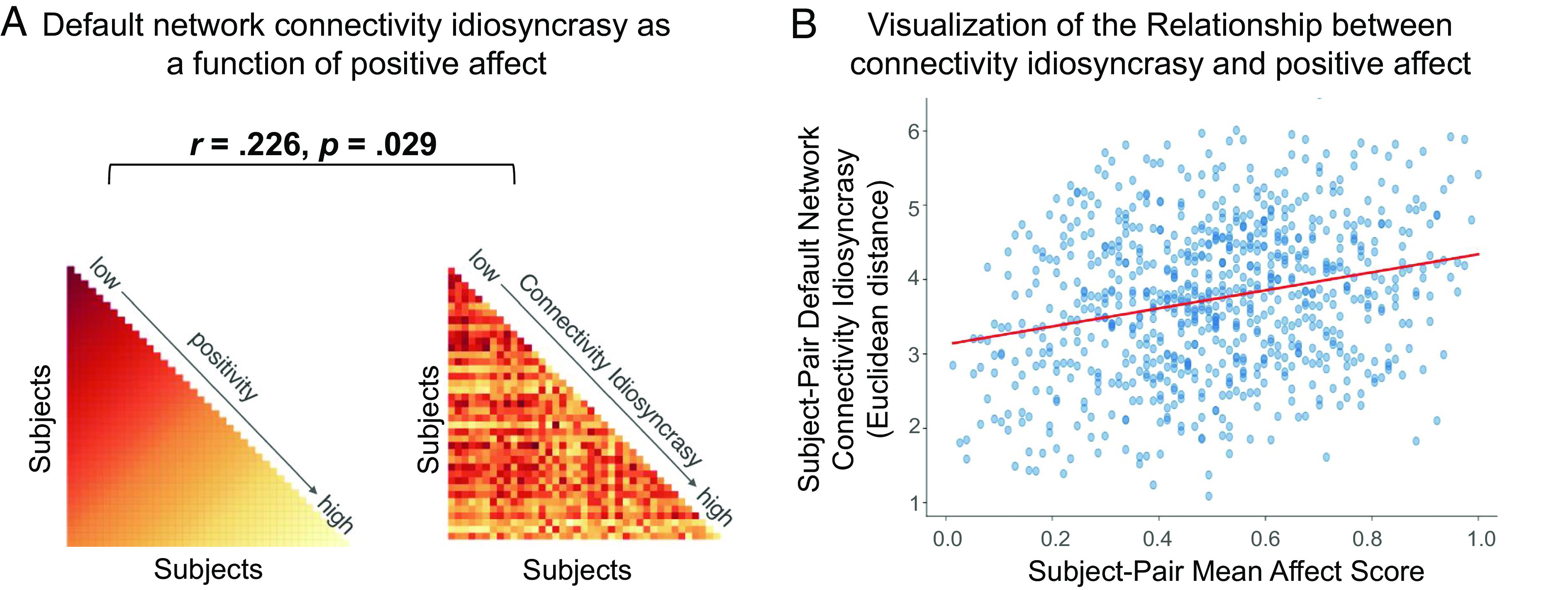
Panel (*A*) shows that the Anna Karenina model significantly predicts the affect in subjects’ memories, with highly negative subjects showing similar default network functional connectivity profiles and highly positive subjects showing idiosyncratic default network functional connectivity profiles. Panel (*B*) shows a visualization of the Anna Karenina model result, highlighting that participants with more positive patient memories show more idiosyncratic default network connectivity profiles.

We found evidence in support of the hypothesis that idiosyncratic default network connectivity comes online after observing negative information to help create a more positive reaction. The Anna Karenina model investigating default network functional connectivity during post-patient rest demonstrated a significant and positive relationship with the affect in subjects’ descriptions of the patients’ videos (*r* = 0.226, *P* = 0.029, Mantel permutation test; Fig. 4). This result indicates that subjects with highly negative patient memories show similar default network functional connectivity profiles during post-patient rest, whereas subjects with highly positive memories show idiosyncratic default network functional connectivity patterns during post-patient rest (i.e., their patterns are different from other positive subjects, as well as other negative subjects). Interestingly, subjects’ mean default network functional connectivity (i.e., the strength of their connectivity) did not significantly relate to affect scores (*r* = −0.142, *P* = 0.383). The default network effects we observed are thus likely driven by idiosyncrasy in subjects’ functional connectivity profiles (i.e., in how the regions communicate with one another), as opposed to simply average connectivity strength. The post-patient rest IS-RSA results were replicated when a different functional parcellation was used to characterize the default network (*SI Appendix*), indicating the results are not specific to the parcellation used here.

The Anna Karenina model testing the relationship between default network connectivity and affect in patient descriptions was not significant for the patient video encoding phase (*r* = −0.045*, P* = 0.646; Mantel permutation test) or baseline rest phase (*r* = 0.013, *P* = 0.907, Mantel permutation test). Parallel analyses testing a link between the affect in science video descriptions and default network connectivity dissimilarity during 1) science encoding or 2) post-science rest were also non-significant (science encoding *r* = −0.018, *P* = 0.858, Mantel permutation test; post-science-rest *r* = −0.065, *P* = 0.511, Mantel permutation test).

Following other naturalistic fMRI studies examining inter-subject (dis)similarities while controlling for other variables (38), we performed partial Mantel tests (39), which assess the correlation between two (dis)similarity matrices while controlling for the effect of an additional (dis)similarity matrix. The Anna Karenina model testing the relationship between default network functional connectivity during post-patient rest and the affect in subjects’ descriptions of the patients’ videos persisted when controlling for the effect of baseline rest (*r* = 0.230 *P* = 0.026, Mantel permutation test), patient video encoding (*r* = 0.280, *P* = 0.006, Mantel permutation test), science video encoding (*r* = 0.201, *P* = 0.048, Mantel permutation test), and post-science rest (*r* = 0.205, *P* = 0.050, Mantel permutation test).

Although affect scores were not significantly correlated with the amount of information recalled, to further assess the specificity of our results, we ran an Anna Karenina model relating subjects’ recall length with their default network functional connectivity profiles from the patient consolidation phase. Specifically, subject pairs were ranked on similarity in recalled length, and then the Anna Karenina analyses were conducted. This model was non-significant (*r* = 0.009, *P* = 0.931, Mantel permutation test), indicating the observed affect results are not likely driven by recall amount more generally. That said, a follow-up, partial Mantel test showed that the Anna Karenina model testing the relationship between default network functional connectivity during post-patient rest and the affect in subjects’ descriptions of the patients’ videos does not remain significant when recall length is controlled for (*r* = 0.030, *P* = 0.370, Mantel permutation test). This is likely due to the fact that although recall length is not significantly correlated with affect, it was positively related.

The Anna Karenina model was not significant when examining connectivity between limbic regions, another potential mechanism underlying these broaden-and-build effects. That is, given the emotional nature of the videos, and the reliance of sentiment analysis on affective words, it is possible that regions in the limbic system would exhibit idiosyncrasy in addition to the default network. To check for this, we ran our analyses in limbic regions associated with affective responding [dorsal anterior cingulate cortex (dACC), anterior insula (AI), and amygdala and separately, dACC, AI, amygdala, and nucleus accumbens (NAc)]. The Anna Karenina model was not significant during any phase for the limbic region analysis (*r*’s < |0.137| *P*’s > 0.210, Mantel permutation tests). Moreover, a follow-up, partial Mantel test demonstrated that the Anna Karenina model testing the relationship between default network functional connectivity during post-patient rest and the affect in subjects’ descriptions of the patients’ videos persisted when controlling for the effect of limbic region functional connectivity (*r* when limbic regions include NAc = 0.228, *P* = 0.013, Mantel permutation test; *r* when limbic regions do not include NAc = 0.230, *P* = 0.013; Mantel permutation test). In other words, the relationship between default network functional connectivity and affect persists above and beyond any relationship between limbic region functional connectivity and affect. Overall, our results converge to suggest that idiosyncratic default network connectivity during offline processes after a negative experience open to interpretation helps generate a more positive lens on the event.

### Default Network Idiosyncrasy Occurring Early in Post-Patient Rest Predicts More Positive Recall: Temporal IS-RSA Results.

**Fig. 5.**
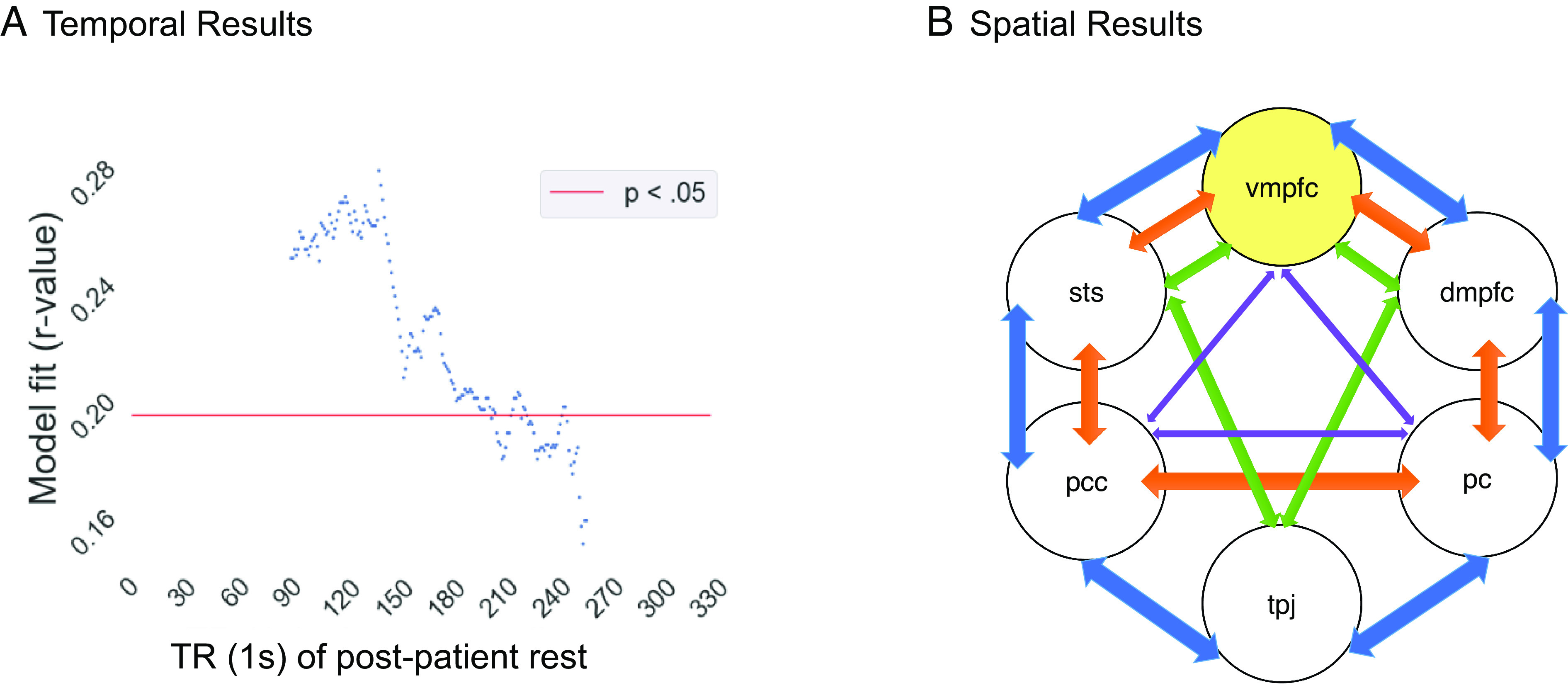
Panel (*A*) shows results from a sliding window analysis demonstrating that the Anna Karenina model is strongest and significant during earlier (vs. later) portions of the post-patient rest phase. Panel (*B*) shows a schematic of the subsetting method. The VMPFC is the only ROI that exists in all network subsets of sizes 3 to 6 that exhibit the Anna Karenina effect during post-patient rest. Network edge colors indicate subset size.

So far, results suggest idiosyncratic default network connectivity during rest after encoding negative information that is open to interpretation corresponds with seeing the good in the bad. We next wanted to know when, over the course of the 6-min rest scan, this effect is most pronounced. Do the subjects who engage idiosyncratic default network responding use the full 6 min of rest to do so? Or does idiosyncrasy happen fairly quickly after encoding to help generate a positive lens? To answer these questions, we investigated whether the relationship between default network functional connectivity and affect is particularly salient during certain periods of the offline processing after listening to the patients’ negative experiences. First, we conducted our analyses separately on connectivity patterns during the first and second halves of the post-patient rest phase, finding significant effects only during the first half (IS-RSA *r*_first_half_ = 0.232, *p*_first_half_ = 0.031, Mantel permutation test; IS-RSA *r*_second_half_ = 0.139, *p*_second_half_ = 0.196, Mantel permutation test). Going one step further, we employed a sliding window approach, with each window still approximately half the duration of the consolidation phase (i.e., 3 min), to more precisely quantify when during rest the Anna Karenina model is most meaningful. To this end, the Anna Karenina model was tested across 3-min time windows starting at t = 0 and shifting by 1 s each time. For example, the first five windows were 1s-180s, 2s-181s, 3s-182s, 4s-183s, and 5s-184s. We found a roughly linear reduction in our model fit, though it included a peak effect for the 50s-220s window before sharply dropping to non-significance (Fig. 5*A*). These results suggest that idiosyncratic default network responses occur relatively quickly during offline processes after a negative experience to help generate a more positive lens on the event.

### Default Network Idiosyncrasy Driven by the VMPFC during Post-Patient Rest Predicts More Positive Recall: ROI-Specific IS-RSA Results.

Prior work suggests that the VMPFC portion of the default network may be particularly key in idiosyncratically generating positive affect in the face of negative events (22, 40). To assess this possibility, we next probed which ROIs in the default network were driving our results. Specifically, we tested which ROIs’ exclusion from the network, individually or along with other ROIs, dissolved our results. To find this, we repeated our IS-RSAs with a default network region “subsetting method,” i.e., we considered a default network subset of size 5 where 1 of the 6 ROIs is dropped from the analyses, then a subset of size 4 where every possible pair of ROIs is dropped from the analyses, then a subset of size 3 where every possible triad of ROIs is dropped from the analyses. Fig. 5*B* shows a schematic of the subsetting method. Our analyses yielded VMPFC as the only ROI that was necessary in all of the subsets that showed significant effects. Specifically, among the three 5-ROI subsets, three 4-ROI subsets and two 3-ROI subsets that fit our model, only the VMPFC showed a consistent significant presence (*r*’s > 0.191, *P*’s < 0.036, Mantel permutation tests). Furthermore, in line with our findings that idiosyncrasies emerge particularly during early rest, we find that this necessity of the VMPFC persists even when analyzing the first half of the post-patient rest period (one 5-ROI subset, three 4-ROI subsets, and one 3-ROI subset; *r*’s > 0.217, *P*’s < 0.039, Mantel permutation tests). These results suggest the default network may require the VMPFC to idiosyncratically generate positive affect in response to negative information.

## Discussion

How, where in the brain, and when are some people able to see the good in the bad? Answering this question is critical to developing a complete scientific model of resiliency and may offer insight into how to intervene to generate optimism in response to negative events. We found that idiosyncratic connectivity in the brain’s default network during rest after exposure to negative information predicts more positive reactions. The findings add critical support for the broaden-and-build theory of positive emotion, which posits that idiosyncratic cognitive processes may help “undo” negative reactions to generate positivity (6). Previously, it was difficult to demonstrate support for this prediction, in part, due to the limited ways to measure idiosyncrasy. To overcome this barrier, we capitalized on a data analytic approach designed to detect idiosyncrasy between participants. We identified that in response to negative information, 1) post-encoding rest is a key moment and 2) the default network is a key brain system in which homogenous responses correspond with negative affect, whereas diversified responses correspond with positive affect.

The results are quite specific to idiosyncratic default network connectivity during post-encoding rest. The Anna Karenina model, which tested the possibility that people who described the videos negatively vs. positively may show different functional connectivity profiles, was significant for default network regions, but not limbic regions, and only during the post-patient rest phase. In fact, follow-up partial Mantel tests demonstrated that post-patient rest results in the default network persisted when controlling for the (null) effects 1) observed during the other phases of the experiment and 2) in limbic regions. Significant effects after controlling for baseline rest rule out the possibility that results are driven by persistent, trait-level differences in default network functional connectivity. Significant effects after controlling for patient encoding further rules out the possibility that post-patient rest results are redundant with differences in perceptual processing during encoding. Significant effects after controlling for science encoding and post-science rest phases further points to the specificity of the results to seeing the good in the bad when there is room for subjective interpretation. Further, significant effects after controlling for limbic regions suggest that while affect may be modulated by these regions, idiosyncratic processing of subjective, affective stimuli are governed by the default network. Moreover, subjects’ mean default network functional connectivity during post-encoding rest was also unrelated to memory affect, further suggesting that results pertain to inter-subject (dis)similarities in functional connectivity patterns. Prior work has shown that processes during post-encoding rest play a causal role in subsequent perceptual and episodic learning and memory, above and beyond encoding effects (28, 41–44). Our results complement these findings, demonstrating that post-encoding processes also play an important role in socioemotional memory formation.

The amount of information written in response to memory prompts (i.e., recall length) was not significantly correlated with affect scores. Additionally, an Anna Karenina model testing whether idiosyncratic default network connectivity predicted recall length was also not significant. That said, it is noteworthy that the relationship between idiosyncratic default network connectivity and affect scores did not remain significant after controlling for recall length, hinting that some shared variance across these metrics explains the results. Indeed, previous research has shown that affective states, especially positive ones, fuel expression and information sharing (45, 46). In our case, positive reactions may fuel writing more lengthy recollections of the videos. Thus, while the default network post-encoding rest period is important for seeing the good in the bad, more research is needed to fully understand how post-encoding mechanisms may link to positive affect and information sharing.

It is noteworthy that while we did not observe a relationship between inter-subject differences during video watching and memory affect, previous research has shown inter-subject differences can occur during encoding. For example, priming and trait biases have been leveraged to evoke individual differences in the default network while viewing ambiguous (19) and polarizing (18) stimuli, respectively. Highly emotional content has also been used to show that negative emotions correspond with greater inter-subject neural synchrony (24). However, the optimal trade-off between a stimulus’ ability to elicit inter-subject, individual differences in neural responding vs. synchronous responding across subjects is unknown (12). Our study uses stimuli which evokes synchrony during encoding while successfully eliciting individual differences only during the post-encoding phase. This may be because the patient videos are overall predominantly negative and we did not recruit different groups of participants who were slated to respond to the videos differently, as is the case, for example, among political partisans from different groups. This further points to the potential importance of post-encoding rest in spontaneously generating individual differences that may not emerge immediately during encoding. Future work is needed to determine whether inter-subject variability during encoding, if generated, can carry over to post-encoding rest, and what the individual contributions of encoding and post-encoding phases are on idiosyncratic cognitive processes. Regardless, our results are consistent with other work indicating that post-encoding processes explain unique variance in subsequent memory (47).

The results also speak to the possibility that inter-subject differences in the default network emerge particularly strongly in response to socioemotional events. Inter-subject differences in neural responding are thought to reflect “subjective construal,” or the way each of us as an individual interprets the world (48). Thus, these differences may occur in response to stimuli that are open to interpretation (17, 49) which are encountered a lot more in social, relative to non-social, situations. For example, in contrast to visual stimuli that portray “the facts on the ground,” other people’s affect, intentions, and beliefs cannot be overtly seen and instead must be interpreted. In fact, when a large group of participants from the human connectome project observed the same animations of shapes moving around a screen, the more certain participants were that the shapes conveyed interpersonal interactions, the more strongly they engaged default network regions [as well as some regions outside of the default network (50)]. Here, we found that inter-subject, default network effects in seeing the good in the bad occurred after hearing patients discuss their experience with cystic fibrosis, but not in response to hearing about the biology of cystic fibrosis. This was the case even though participants did not express significantly dissimilar amounts of affect when describing both types of videos. This observation, paired with prior work finding that default network regions preferentially consolidate social (vs. non-social) information during rest (27), speaks to the particularly important role of the default network in generating subjective interpretations of the social world.

Two sets of follow-up analyses revealed additional insight into the spatio-temporal basis of these effects. First, temporal analyses revealed that our inter-subject results emerge during early post-patient rest. The Anna Karenina model was significant during the first, but not second, half of the post-patient rest phase and sliding window analyses further revealed that the effect occurs in the first ~3.5 min of rest. The temporal immediacy may be driven by multiple factors. One possibility is that most post-encoding effects occur during early rest, reflecting a type of recency effect in mind wandering. Alternatively, it is possible that specifically interpersonal information, as was conveyed in the patient videos, may be temporally prioritized by the brain during post-encoding processes. It has been argued that goal-relevant information may be “tagged” for prioritized memory consolidation at rest (51). Given that 1) humans have a strong, endogenous goal to feel connected to others (52) and 2) default network regions engage “by default” during rest (53), interpersonal information may be prioritized during post-encoding rest, with individual differences in affect codified quickly. Future work that manipulates 1) when rest occurs after interpersonal interactions (e.g., immediately vs. delayed) and 2) the duration of rest will clarify which of these competing possibilities best explains the temporal immediacy of interpersonal learning and memory.

The second set of follow-up analyses revealed VMPFC as a key player driving the default network results. We used a “subsetting” approach to the IS-RSA default network post-encoding rest analyses in which we considered how removing an ROI impacts results. This approach showed VMPFC as the only ROI that was necessary for the IS-RSA results. VMPFC is associated with generating affective interpretations, particularly positive ones (40). Moreover, recent work implicates VMPFC in supporting idiosyncratic responses (54), particularly in response to naturalistic social stimuli (23). Our results therefore nicely complement and extend prior research on VMPFC, highlighting that this region plays a key role in asymmetrically predicting affect, with homogenous connectivity with default network regions predicting negative affect and idiosyncratic connectivity with default network regions predicting positive affect.

The VMPFC is part of the dopaminergic system, which is critical to reward and motivational processing. Interestingly, one recent study found that engaging the VMPFC during post-encoding rest helps subjects unlearn negative associations, an effect amplified by psychopharmacological increases in dopamine (55). Future work can examine whether the relationship between idiosyncratic, post-encoding VMPFC-default network connectivity is thus mediated by dopamine. Additionally, the VMPFC has bidirectional links to brain regions that generate the physiological stress response (e.g., hypothalamus), and positive affect has also been shown to reduce stress (56, 57). It may thus also be fruitful to assess the role of idiosyncratic, post-encoding VMPFC-default network connectivity in dampening physiological markers of stress.

More broadly, the results hint at a way to think about the neurocognitive mechanisms supporting resilience and the “broaden and build” theory of positive emotion. In terms of resilience, extensive psychological research suggests that finding positive meaning in response to negative situations, such as a stressor bringing people together, promotes mental and physical health (58). When caretakers find the benefits of a patient’s experience with a disease, both the patient and caretaker experience better well-being (2, 31–36). Our paradigm mirrors this situation, as our subjects listened to patients with cystic fibrosis share their experience with the diagnosis. This parallel, paired with the fact that prior work found VMPFC increases activity when subjects are explicitly instructed to find positive meaning in response to negative stimuli (22), points to the possibility that idiosyncratic VMPFC responding during consolidation may support the resilient strategy to see the good in the bad. Moreover, the broaden-and-build theory of positive emotion has previously suggested that idiosyncratic cognitive processes are associated with positive affect, but with limited insight into how this occurs. Our results update this literature, showing that the idiosyncratic thoughts that promote positive affect may occur spontaneously (i.e., without instruction) during post-encoding rest. The present work generates the prediction that inducing idiosyncratic thinking directly after a negative event may help people walk away with a more optimistic view.

## Limitations

There are three important limitations to this work. First, although our neural results are statistically robust, it is unclear whether the post-encoding effects we observed reflect conscious reappraisals of the patients’ situations vs. processes inaccessible to conscious awareness. Relatedly, although our text-based approach to measuring affect is objective, it is unclear whether the results line up with participants’ subjective experience of the stimuli. Future research that combines experience sampling with post-encoding rest periods may provide insight into these possibilities. Second, although our ROIs were defined by independent data, they are also relatively large. We prioritized objectively defined default network brain regions that are also functionally relevant to psychological constructs. For this reason, we employed the k = 50 whole brain parcellation that used k-means clustering to isolate meta-analytic coactivations (59) from Neurosynth (60). As a result, it is unclear which precise subclusters of voxels within each ROI are particularly key to our findings. Future research with more targeted anatomical hypotheses may reveal the fine-grained patterns within the default network that contribute to seeing the good in the bad. Third, we cannot determine whether the positive affect in participants’ responses corresponds with greater trait optimism more generally. The present work aims to answer how people are able to “see the good in the bad,” which is why we focused on positive sentiment in response to negative content, rather than trait optimism. That said, past work has linked trait optimism with positive affect (61–63). Future work can examine the psychological traits, such as optimism, that may modulate post-encoding default network responding.

## Conclusion

It is impossible to avoid negative experiences in life, which is why effective coping strategies are key to protecting mental and physical health. One well-known coping strategy is positive thinking (64), including the tendency to stay positive in the face of life’s challenges (1–5). Yet, to date, the underlying mechanisms that allow some people to spontaneously employ this resilient strategy have been unclear and speculative. We found that idiosyncratic responses by the brain’s default network, when we rest immediately after hearing about life’s hardships, explain why some of us react with optimism while others with despair. People who can see negative situations through rose-colored glasses may be able to do so, in part, by wearing their own, unique lens after the event.

## Methods

### Participants.

Forty right-handed subjects (26 female; mean age = 29 y, SD = 11, 65% white; 23% Asian; 8% Hispanic) completed this study. Subjects either received $20 per hour of participation or were awarded course credit for completing the experiment. The study protocol was approved by the Dartmouth College IRB. All participants provided informed consent. The data used here have been reported on in prior work (65), though notably all analyses reported here are orthogonal to those previously reported.

### Procedures.

During the rest scans, subjects saw a blank screen and rested while awake. During the patient encoding scans, subjects watched four videos in which people with cystic fibrosis discuss their experiences with the disease, with each patient video lasting approximately 4 min. To determine which patient videos to use in the fMRI session, we initially had an online sample of participants (N = 197) observe six patient videos and continuously rate on a 0 to 10 scale how much empathy they felt for the patients while watching their videos. We then selected the four videos that induced the greatest empathy: Video 1 mean = 7.86; Video 2 mean = 7.32; Video 3 mean = 7.23; Video 4 mean = 6.97. This approach helps ensure that the patient videos can induce a relevant emotional response. During the science encoding, subjects watched four, approximately 4-min Khan Academy videos describing the biology of cystic fibrosis. The order of patient and science encoding was counterbalanced across subjects. Subjects also completed an anatomical scan which was used for fMRI image processing.

### Sentiment Analysis of Memory Recall.

Directly after the fMRI scanning, subjects wrote down descriptions of the videos. Each subject’s typed responses to the four patient videos were combined into one paragraph per subject, and the recall of the four science videos were combined into another paragraph per subject. The paragraphs were cleaned to exclude non-alphabets and common “stop-words” (prepositions) as defined by python’s Natural Language ToolKit [NLTK (66)]. Next, we performed sentiment analysis [VADER (37)] on each word, such that negative words were affect scored between −1 and 0 (with more negative words scoring closer to −1), and positive words were affect scored between 0 and 1 (with more positive words scoring closer to 1). Finally, we summed the word affect scores across the entire paragraph to obtain one affect score per subject. We chose to use the sum instead of the average affect score because the variance and skew across summed affect scores showed it to be the more appropriate metric to analyze (summed affect *var* = 3.68, *skew* = 0.135; mean affect *var* = −0.004, *skew* = −0.9). To rule out confounds, we ensured that the affect scores were not significantly correlated with the number of affect words (*r* = 0.096, *P* = 0.554) or number of overall words (*r* = 0.243, *P* = 0.131).

### fMRI Data Acquisition.

Scanning was performed on a Siemens Prisma 3-T Trio. Functional images were acquired using an EPI gradient- echo sequence (2.5 × 2.5 × 2.5 mm voxels, repetition time = 1,000 ms, echo time = 30 ms, 2.5-mm slice thickness, field of view = 24 cm, matrix = 96 × 96, flip angle = 59°, multiband acceleration factor = 4). A T2- weighted structural image was acquired coplanar with the functional images (0.9 × 0.9 × 0.9 mm voxels, repetition time = 2,300 ms, echo time = 2.32 ms, 0.9-mm slice thickness, field of view = 24 cm, matrix = 256 × 256, flip angle = 8°).

### fMRI Preprocessing.

Functional and anatomical brain images were reoriented using SPM and skull-stripped using the Brain Extraction Tool in FSL. Data were preprocessed using FSL. Specifically, data underwent high-pass filtering (0.009 Hz cutoff), motion correction, skull-stripping, spatial smoothing (6 mm radius), and registration to the anatomical image using Boundary-Based Registration. Nuisance variables, which included six standard motion parameters, their derivatives, as well as white matter and cerebrospinal fluid data, were regressed out using GLMs. White matter and cerebrospinal fluid masks were generated from each anatomical image using FSL’s FMRIB’s Automated Segmentation Tool (67). Additionally, to correct for extreme motion, global (average brain) signal and motion scrubbing (volumes with framewise displacement > 0.2 mm) artifacts were regressed out. All analyses are applied to the residual images from this nuisance-variable GLM.

### Neural Time Series Extraction.

We wanted to objectively define default network brain regions while also ensuring that the regions selected are functionally relevant to psychological constructs. For this reason, we used the k = 50 whole brain parcellation that used k-means clustering to isolate meta-analytic coactivations (59) from Neurosynth (60) though see *SI Appendix* for a replication of results using a different parcellation scheme). The parcellation used here includes six parcels that comprise the default network: the ventromedial prefrontal cortex (VMPFC), dorsomedial prefrontal cortex (DMPFC), temporo-parietal junction (TPJ), precuneus (PC), and posterior cingulate cortex functionally combined with posterior TPJ (pcc/pTPJ), and the superior temporal sulcus extending into temporal poles (STS/TP; default network regions depicted in Fig. 2). The whole brain parcellation also includes limbic regions traditionally associated with affective responding: dorsal anterior cingulate cortex (dACC), anterior insula (AI), amygdala, and nucleus accumbens (NAc). We therefore were able to examine functional connectivity (i.e., timecourse correlations reflecting coactivation) for each subject for 1) the default network, 2) limbic regions, and 3) across the whole brain (i.e., all 50 parcels). These and all subsequent analyses were performed on default network regions, as well as separately, brain regions in the limbic system as well as brain regions across the entire brain. We ran limbic region analyses two ways: first, with just the dACC, AI, and amygdala and, second, with these regions as well as the nucleus accumbens (NAc), given its reliable role in positive affect. This two-pronged approach was taken because the dACC, AI, and amygdala are associated with both negative and positive affect (68), whereas the NAc is more consistently associated with positive affect only (69, 70).

For each subject, the z-scored time series of neural activation in each parcel was extracted for each phase of scanning—rest scans and video encoding scans. Following recommendations from prior work (49), we not only excluded the pre- and post-video fixation seconds, but also the first 10 s of the videos themselves, to prevent the onset of videos from inflating inter-subject similarity in connectivity. Similarly, we excluded the first 10 s of the baseline and consolidation phases each (i.e., the first 10 s of each rest period).

### Inter-Subject Similarity in Functional Connectivity.

Before investigating individual differences, we assessed whether as a group, participants show similar functional connectivity profiles. This helps ensure that across subjects, our measure of functional connectivity is reliable before examining its relationship to individual differences in affect. To this end, we first calculated a subject’s default network functional connectivity by computing the Pearson correlation of the neural time series between each default network parcel pair (separately for each experimental phase). Next, we computed the similarity in subjects’ connectivity as the Pearson correlation of connectivity vectors between subject pairs, following a previous protocol on inter-subject similarity in BOLD signal timecourses (71). We examined subjects’ functional connectivity patterns (rather than, for example, a given region’s timecourse) given prior work suggesting 1) offline processing during rest occurs via communication between brain regions (26) and 2) when no stimuli are present as is the case during rest, between-subject dissimilarity in functional connectivity profiles are more interpretable than between-subject dissimilarity in regional timecourses (72–74).

These steps were also taken in a series of follow-up analyses designed to assess the specificity of the results to default network regions. First, with the limbic regions dACC, AI, and amygdala, which created a 3*3 matrix of parcel-pair connectivity per subject, and then separately with the nucleus accumbens (NAc), dACC, AI, and amygdala, which created a 4*4 matrix.

### Testing for Significance of Inter-Subject Similarity in Functional Connectivity in Each Phase and Brain Network.

To test whether, collapsing across individual differences, similarity in inter-subject connectivity was significant for any given phase or network, we computed the *P-*value as the proportion of null distribution values that were greater than the observed median inter-subject connectivity similarity. To generate this null distribution of median inter-subject similarity, we conduct a subject-wise bootstrap with replacement, each time correlating the resultant subjects’ profiles with one another to obtain a new median inter-subject similarity value. The bootstrapping-with-replacement procedure allows for some subjects to be sampled multiple times, introducing off-diagonal values of 1 (since a subject is always perfectly correlated with him or herself); these values are ignored when calculating the new median. This new median is then shifted by the true summary statistic (observed median correlation); this procedure is repeated n = 5,000 times to create a null distribution centered around zero. Finally, we calculate the proportion of null distribution values that are greater than our actual median inter-subject similarity and use that as our significance (*P*) value.

It is noteworthy that bootstrapping is commonly used for generating CIs around an estimator, while permutation tests are commonly used for performing hypothesis tests. However, *P*-values can also be computed using a bootstrap by subtracting the ISC from the null distribution and evaluating the percent of samples from the distribution that are smaller or greater than the observed ISC (75). Thus, in the subject-wise bootstrapping approach, we test the median subject pair similarity against a null distribution which is generated by repeatedly taking the median of subject-level bootstrapped-with-replacement inter-subject matrices. The proportion of null medians greater than observed medians is considered as our *P*-value threshold. Note that this approach is more conservative than the previously described methods (76).

### Inter-Subject Mean Affect Model Creation.

To link individual differences in memory affect to neural activity, we first converted subjects’ affect scores into ranks, such that negative subjects were ranked low and positive subjects were ranked high (range of ranks = 0 to 39 for N = 40). Our Anna Karenina model modeled subject pair’s dissimilarity in functional connectivity as the mean of the pair’s affect ranks, such that the higher the pair’s rank (indicating more positive recall), the greater their dissimilarity in functional connectivity (indicating more idiosyncratic connectivity), and vice versa. We thus obtained our 40*40 inter-subject mean affect model. The Anna Karenina model is depicted in Fig. 3.

### Inter-Subject Representational Similarity Analysis (IS-RSA) with Mantel Tests.

Finally, to test our hypothesized association between positive memories and idiosyncratic connectivity, we first reframed the inter-subject similarity matrices as dissimilarity matrices, with greater dissimilarity reflecting more idiosyncrasies in functional connectivity. To this end, instead of Pearson correlating subjects’ connectivity vectors (which reflects similarity; see “*Inter-Subject Similarity in Functional Connectivity*”), we compute the Euclidean distance (which reflects dissimilarity) between subjects’ z-scored vectors, such that greater distances represented greater dissimilarity (in our data, the greatest Euclidean distance between any pair of z-scored connectivity vectors was 6.485). This analysis follows previous methodology as conducted by Hyon et al. (72). Note that either analysis (Pearson correlation or Euclidean distance) preserves the rank order of subject pairs in terms of their (dis)similarity, and merely flips the language of interpretation. Next, we Spearman correlated our inter-subject mean affect and connectivity dissimilarity matrices (specifically, we only correlated the lower triangles of these symmetric matrices). Here, as is protocol in the Representational Similarity Analysis literature (77), we used Spearman correlations because increase in connectivity dissimilarity may not be linear to the increase in the mean affect of a subject pair. As noted in our inter-subject connectivity similarity methods section above, to determine the statistical significance of a model’s fit, we needed to account for the non-independence in our data: Specifically, each data point (matrix cell) represented a subject pair, and thus each subject was represented in multiple (N-1 = 39) data points. To this end, and consistent with prior work (17), we conducted a non-parametric, Mantel permutation test, wherein we randomly shuffled and reassigned subjects’ functional connectivity vectors 5,000 times, each time correlating the resultant simulated connectivity dissimilarity matrix with our unshuffled mean affect matrix, thus generating a (null) distribution of IS-RSA (correlation) values. To be clear, we shuffle at the subject level, rather than neural (i.e., ROI) level; in other words, each subject’s (intact) functional connectivity vector was relabeled with a different subject’s identity (and therefore mean affect score), to break the expected relationship between individual functional connectivity vectors and affect scores. We then calculated the proportion of times our simulated null correlation value exceeded our observed model-data correlation, yielding the probability that our results were generated by chance. Finally, we compared this probability against a significance threshold of alpha = 0.05 to discern statistical significance.

### Control Analyses with Partial Mantel Tests.

Next, to test the specificity of our results to default network connectivity during post-patient rest, we performed partial Mantel tests, which essentially is a Mantel test as described above, but between residual inter-subject mean affect and connectivity dissimilarity matrices, after covariations with confounding experimental phases and brain networks were regressed out. For this, we used the “vegan” package (https://cran.r-project.org/web/packages/vegan/) implementation of the partial Mantel test (39). Specifically, we conducted six partial Mantel tests, one for ruling out default network connectivity during each of the other four phases, and one each for ruling out limbic region connectivity, with and without NAc, during post-patient rest, thus totaling 6. Each of the six partial Mantel tests includes two linear regression models predicting the connectivity dissimilarity for the given covarying condition (example 1: limbic region dissimilarity during post-patient rest; example 2: default network dissimilarity during baseline rest). The predictor for one model was the inter-subject mean affect and for the other was the inter-subject connectivity dissimilarity. The errors from each model give us residual inter-subject mean affect and connectivity dissimilarity, respectively, which we then performed a Mantel test on (i.e., comparing their rank correlation to a null distribution generated via subject-wise permutation).

## Supplementary Material

Appendix 01 (PDF)

## Data Availability

Code and data are available at https://github.com/siyer7/default_network-socioaffective-memory-variability.

## References

[1] Y. Kim, R. Schulz, C. S. Carver, Benefit-finding in the cancer caregiving experience. Psychosom Med. **69**, 283–291 (2007). 17420443 10.1097/PSY.0b013e3180417cf4

[2] M. L. Chung , Linkage of optimism with depressive symptoms among the stroke survivor and caregiver dyads at 2 years post stroke: Dyadic mediation approach. J. Cardiovasc. Nurs. (2022), 10.1097/JCN.0000000000000920. 35467560

[3] C. vanOyen Witvliet, L. M. Root Luna, R. D. Vlisides-Henry, G. D. Griffin, Consecutive reappraisal strategies strengthen and sustain empathy and forgiveness: Utilizing compassion and benefit finding while holding offenders accountable. J. Posit. Psychol. **15**, 362–372 (2020).

[4] M. E. McCullough, L. M. Root, A. D. Cohen, Writing about the benefits of an interpersonal transgression facilitates forgiveness. J. Consult. Clin. Psychol. **74**, 887–897 (2006). 17032093 10.1037/0022-006X.74.5.887

[5] K. M. Brethel-Haurwitz, M. Stoianova, A. A. Marsh, Empathic emotion regulation in prosocial behaviour and altruism. Cogn. Emot. **34**, 1532–1548 (2020). 32576078 10.1080/02699931.2020.1783517

[6] B. L. Fredrickson, The broaden-and-build theory of positive emotions. Philos. Trans. R Soc. Lond. B Biol. Sci. **359**, 1367–1378 (2004). 15347528 10.1098/rstb.2004.1512 PMC1693418

[7] B. A. Nijstad, C. K. W. De Dreu, E. F. Rietzschel, M. Baas, The dual pathway to creativity model: Creative ideation as a function of flexibility and persistence. Eur. Rev. Soc. Psychol. **21**, 34–77 (2010).

[8] A. M. Isen, M. M. Johnson, E. Mertz, G. F. Robinson, The influence of positive affect on the unusualness of word associations. J. Pers. Soc. Psychol. **48**, 1413–1426 (1985). 4020605 10.1037//0022-3514.48.6.1413

[9] S. A. Barnes , Modulation of ventromedial orbitofrontal cortical glutamatergic activity affects the explore-exploit balance and influences value-based decision-making. Cereb. Cortex **33**, 5783–5796 (2022), 10.1093/cercor/bhac459. PMC10183731 36472411

[10] R. van Dooren, R. de Kleijn, B. Hommel, Z. Sjoerds, The exploration-exploitation trade-off in a foraging task is affected by mood-related arousal and valence. Cogn. Affect. Behav. Neurosci. **21**, 549–560 (2021). 34086199 10.3758/s13415-021-00917-6 PMC8208924

[11] A. S. Heller , Association between real-world experiential diversity and positive affect relates to hippocampal–striatal functional connectivity. Nat. Neurosci. **23**, 800–804 (2020). 32424287 10.1038/s41593-020-0636-4 PMC9169417

[12] E. S. Finn , Idiosynchrony: From shared responses to individual differences during naturalistic neuroimaging. Neuroimage **215**, 116828 (2020). 32276065 10.1016/j.neuroimage.2020.116828 PMC7298885

[13] Y. Yeshurun, M. Nguyen, U. Hasson, The default mode network: Where the idiosyncratic self meets the shared social world. Nat. Rev. Neurosci. **22**, 181–192 (2021). 33483717 10.1038/s41583-020-00420-w PMC7959111

[14] A. B. Satpute, K. A. Lindquist, The default mode network’s role in discrete emotion. Trends Cogn. Sci. **23**, 851–864 (2019). 31427147 10.1016/j.tics.2019.07.003 PMC7281778

[15] M. A. Thornton, D. I. Tamir, People represent mental states in terms of rationality, social impact, and valence: Validating the 3d Mind Model. Cortex **125**, 44–59 (2020). 31962230 10.1016/j.cortex.2019.12.012 PMC7093241

[16] L. J. Chang, P. J. Gianaros, S. B. Manuck, A. Krishnan, T. D. Wager, A sensitive and specific neural signature for picture-induced negative affect. PLoS Biol. **13**, e1002180 (2015). 26098873 10.1371/journal.pbio.1002180 PMC4476709

[17] E. S. Finn, P. R. Corlett, G. Chen, P. A. Bandettini, R. T. Constable, Trait paranoia shapes inter-subject synchrony in brain activity during an ambiguous social narrative. Nat. Commun. **9**, 2043 (2018). 29795116 10.1038/s41467-018-04387-2 PMC5966466

[18] Y. C. Leong, J. Chen, R. Willer, J. Zaki, Conservative and liberal attitudes drive polarized neural responses to political content. Proc. Natl. Acad. Sci. U.S.A. **117**, 27731–27739 (2020). 33082227 10.1073/pnas.2008530117 PMC7959490

[19] J. M. van Baar, D. J. Halpern, O. FeldmanHall, Intolerance to uncertainty modulates neural synchrony between political partisans. bioRxiv [Preprint] (2020). 10.1101/2020.10.28.358051 (Accessed 29 October 2020).

[20] E. Simony , Dynamic reconfiguration of the default mode network during narrative comprehension. Nat. Commun. **7**, 12141 (2016). 27424918 10.1038/ncomms12141 PMC4960303

[21] T. W. Broom, J. L. Stahl, E. E. C. Ping, D. D. Wagner, They saw a debate: Political polarization is associated with greater multivariate neural synchrony when viewing the opposing candidate speak. J. Cogn. Neurosci. **35**, 60–73 (2022). 35802592 10.1162/jocn_a_01888

[22] B. P. Doré , Finding positive meaning in negative experiences engages ventral striatal and ventromedial prefrontal regions associated with reward valuation. J. Cogn. Neurosci. **29**, 235–244 (2017). 27626229 10.1162/jocn_a_01041

[23] L. J. Chang , Endogenous variation in ventromedial prefrontal cortex state dynamics during naturalistic viewing reflects affective experience. Sci. Adv. **7**, eabf7129 (2021), 10.1126/sciadv.abf7129. 33893106 PMC8064646

[24] L. Nummenmaa , Emotions promote social interaction by synchronizing brain activity across individuals. Proc. Natl. Acad. Sci. U.S.A. **109**, 9599–9604 (2012). 22623534 10.1073/pnas.1206095109 PMC3386135

[25] M. E. Speer, S. Ibrahim, D. Schiller, M. R. Delgado, Finding positive meaning in memories of negative events adaptively updates memory. Nat. Commun. **12**, 6601 (2021). 34782605 10.1038/s41467-021-26906-4 PMC8593143

[26] A. Tambini, L. Davachi, Awake reactivation of prior experiences consolidates memories and biases cognition. Trends Cogn. Sci. **23**, 876–890 (2019). 31445780 10.1016/j.tics.2019.07.008 PMC6907733

[27] M. L. Meyer, L. Davachi, K. N. Ochsner, M. D. Lieberman, Evidence that default network connectivity during rest consolidates social information. Cereb Cortex. **29**, 1910–1920 (2019). 29668862 10.1093/cercor/bhy071

[28] E. J. Wamsley, Offline memory consolidation during waking rest. Nat. Rev. Psychol. **1**, 441–453 (2022).

[29] K. L. Hoffman, B. L. McNaughton, Coordinated reactivation of distributed memory traces in primate neocortex. Science **297**, 2070–2073 (2002). 12242447 10.1126/science.1073538

[30] É. Slattery, J. McMahon, S. Gallagher, Optimism and benefit finding in parents of children with developmental disabilities: The role of positive reappraisal and social support. Res. Dev. Disabil. **65**, 12–22 (2017). 28432893 10.1016/j.ridd.2017.04.006

[31] K. I. Pakenham, The positive impact of multiple sclerosis (MS) on carers: Associations between carer benefit finding and positive and negative adjustment domains. Disabil. Rehabil. **27**, 985–997 (2005). 16096252 10.1080/09638280500052583

[32] A. Nieto , The distinctive role of grounded optimism and resilience for predicting burnout and work engagement: A study in professional caregivers of older adults. Arch. Gerontol. Geriatr. **100**, 104657 (2022). 35182990 10.1016/j.archger.2022.104657

[33] K. L. Rand, A. M. Shea, “Optimism within the context” in The Oxford Handbook of Positive Psychology and Disability, M. L. Wehmeyer, Ed. (Oxford University Press, 2013), **vol. 48**.

[34] S. Byra, R. Zubrzycka, P. W’ojtowicz, Sense of stress and posttraumatic growth in mothers of children with cystic fibrosisthe moderating role of resilience. J. Dev. Behav. Pediatr. **42**, e8–e14 (2021). 34334723 10.1097/DBP.0000000000000967

[35] Y. Chang, H.-J. Chan, Optimism and proactive coping in relation to burnout among nurses. J. Nurs. Manag. **23**, 401–408 (2015). 24112222 10.1111/jonm.12148

[36] K. W. Luthans, S. A. Lebsack, R. R. Lebsack, Positivity in healthcare: Relation of optimism to performance. J. Health Organ. Manag. **22**, 178–188 (2008). 18700527 10.1108/14777260810876330

[37] C. Hutto, E. Gilbert, VADER: A parsimonious rule-based model for sentiment analysis of social media text. ICWSM **8**, 216–225 (2014).

[38] C. Sava-Segal, C. Richards, M. Leung, E. S. Finn, Individual differences in neural event segmentation of continuous experiences. Cereb. Cortex **33**, 8164–8178 (2023). 36994470 10.1093/cercor/bhad106 PMC10321113

[39] P. E. Smouse, J. C. Long, R. R. Sokal, Multiple regression and correlation extensions of the mantel test of matrix correspondence. Syst. Zool. **35**, 627–632 (1986).

[40] M. Roy, D. Shohamy, T. D. Wager, Ventromedial prefrontal-subcortical systems and the generation of affective meaning. Trends Cogn. Sci. **16**, 147–156 (2012). 22310704 10.1016/j.tics.2012.01.005 PMC3318966

[41] J. W. Bang, D. Milton, Y. Sasaki, T. Watanabe, D. Rahnev, Post-training TMS abolishes performance improvement and releases future learning from interference. Commun. Biol. **2**, 320 (2019). 31482139 10.1038/s42003-019-0566-4 PMC6711956

[42] A. Tambini, M. D’Esposito, Causal contribution of awake post-encoding processes to episodic memory consolidation. Curr. Biol. **30**, 3533–3543.e7 (2020). 32735812 10.1016/j.cub.2020.06.063 PMC7511431

[43] K. Brokaw , Resting state EEG correlates of memory consolidation. Neurobiol. Learn. Mem. **130**, 17–25 (2016). 26802698 10.1016/j.nlm.2016.01.008

[44] M. Craig, M. Dewar, M. A. Harris, S. Della Sala, T. Wolbers, Wakeful rest promotes the integration of spatial memories into accurate cognitive maps. Hippocampus **26**, 185–193 (2016). 26235141 10.1002/hipo.22502

[45] C. Wang, Z. Zhou, X.-L. Jin, Y. Fang, M. K. O. Lee, The influence of affective cues on positive emotion in predicting instant information sharing on microblogs: Gender as a moderator. Inf. Process Manag. **53**, 721–734 (2017).

[46] S. Stieglitz, L. Dang-Xuan, Emotions and information diffusion in social media—sentiment of microblogs and sharing behavior. J. Manag. Inform. Syst. **29**, 217–248 (2013).

[47] M. J. Gruber, M. Ritchey, S.-F. Wang, M. K. Doss, C. Ranganath, Post-learning hippocampal dynamics promote preferential retention of rewarding events. Neuron **89**, 1110–1120 (2016). 26875624 10.1016/j.neuron.2016.01.017 PMC4777629

[48] D. W. Griffin, L. Ross, “Subjective construal, social inference, and human misunderstanding” in Advances in Experimental Social Psychology, M. P. Zanna, Ed. (Academic Press, 1991), pp. 319–359.

[49] S. A. Nastase, V. Gazzola, U. Hasson, C. Keysers, Measuring shared responses across subjects using intersubject correlation. Soc. Cogn. Affect. Neurosci. **14**, 667–685 (2019). 31099394 10.1093/scan/nsz037 PMC6688448

[50] R. S. Varrier, E. S. Finn, Seeing social: A neural signature for conscious perception of social interactions. J. Neurosci. **42**, 9211–9226 (2022). 36280263 10.1523/JNEUROSCI.0859-22.2022 PMC9761685

[51] E. T. Cowan, A. C. Schapiro, J. E. Dunsmoor, V. P. Murty, Memory consolidation as an adaptive process. Psychon. Bull. Rev. **28**, 1796–1810 (2021). 34327677 10.3758/s13423-021-01978-x

[52] R. F. Baumeister, M. R. Leary, The need to belong: Desire for interpersonal attachments as a fundamental human motivation. Psychol. Bull. **117**, 497–529 (1995). 7777651

[53] M. E. Raichle, The brain’s default mode network. Annu. Rev. Neurosci. **38**, 433–447 (2015). 25938726 10.1146/annurev-neuro-071013-014030

[54] M. D. Lieberman, M. A. Straccia, M. L. Meyer, M. Du, K. M. Tan, Social, self, (situational), and affective processes in medial prefrontal cortex (MPFC): Causal, multivariate, and reverse inference evidence. Neurosci. Biobehav. Rev. **99**, 311–328 (2019). 30610911 10.1016/j.neubiorev.2018.12.021

[55] A. M. V. Gerlicher, O. Tüscher, R. Kalisch, Author Correction: Dopamine-dependent prefrontal reactivations explain long-term benefit of fear extinction. Nat. Commun. **10**, 471 (2019). 30674882 10.1038/s41467-019-08399-4 PMC6344470

[56] L. Koban, P. J. Gianaros, H. Kober, T. D. Wager, The self in context: Brain systems linking mental and physical health. Nat. Rev. Neurosci. **22**, 309–322 (2021). 33790441 10.1038/s41583-021-00446-8 PMC8447265

[57] N. I. Eisenberger, S. W. Cole, Social neuroscience and health: Neurophysiological mechanisms linking social ties with physical health. Nat. Neurosci. **15**, 669–674 (2012). 22504347 10.1038/nn.3086

[58] V. S. Helgeson, K. A. Reynolds, P. L. Tomich, A meta-analytic review of benefit finding and growth. J. Consult. Clin. Psychol. **74**, 797–816 (2006). 17032085 10.1037/0022-006X.74.5.797

[59] A. de la Vega, L. J. Chang, M. T. Banich, T. D. Wager, T. Yarkoni, Large-scale meta-analysis of human medial frontal cortex reveals tripartite functional organization. J. Neurosci. **36**, 6553–6562 (2016). 27307242 10.1523/JNEUROSCI.4402-15.2016 PMC5015787

[60] T. Yarkoni, R. A. Poldrack, T. E. Nichols, D. C. Van Essen, T. D. Wager, Large-scale automated synthesis of human functional neuroimaging data. Nat. Methods. **8**, 665–670 (2011). 21706013 10.1038/nmeth.1635 PMC3146590

[61] S. C. Segerstrom, S. E. Sephton, Optimistic expectancies and cell-mediated immunity: The role of positive affect. Psychol. Sci. **21**, 448–455 (2010). 20424083 10.1177/0956797610362061 PMC3933956

[62] J. K. Boehm, Y. Chen, D. R. Williams, C. Ryff, L. D. Kubzansky, Unequally distributed psychological assets: Are there social disparities in optimism, life satisfaction, and positive affect? PLoS One. **10**, e0118066 (2015). 25671665 10.1371/journal.pone.0118066 PMC4324648

[63] A. Steptoe, S. Dockray, J. Wardle, Positive affect and psychobiological processes relevant to health. J. Pers. **77**, 1747–1776 (2009). 19796062 10.1111/j.1467-6494.2009.00599.x PMC2787693

[64] S. E. Taylor, M. E. Kemeny, G. M. Reed, J. E. Bower, T. L. Gruenewald, Psychological resources, positive illusions, and health. Am. Psychol. **55**, 99–109 (2000). 11392870 10.1037//0003-066x.55.1.99

[65] E. Collier, M. L. Meyer, Memory of others’ disclosures is consolidated during rest and associated with providing support: Neural and linguistic evidence. J. Cogn. Neurosci. **32**, 1672–1687 (2020). 32379001 10.1162/jocn_a_01573

[66] S. Bird, NLTK: The natural language toolkit (2006) (cited 22 Mar 2023). Available: https://aclanthology.org/P06-4018.pdf.

[67] Y. Zhang, M. Brady, S. Smith, Segmentation of brain MR images through a hidden Markov random field model and the expectation-maximization algorithm. IEEE Trans. Med. Imag. **20**, 45–57 (2001). 10.1109/42.906424 11293691

[68] W. A. Cunningham, T. Brosch, Motivational salience: Amygdala tuning from traits, needs, values, and goals. Curr. Dir. Psychol. Sci. **21**, 54–59 (2012).

[69] B. Knutson, C. M. Adams, G. W. Fong, D. Hommer, Anticipation of increasing monetary reward selectively recruits nucleus accumbens. J. Neurosci. **21**, RC159 (2001). 11459880 10.1523/JNEUROSCI.21-16-j0002.2001 PMC6763187

[70] M. Koch, A. Schmid, H. U. Schnitzler, Pleasure-attenuation of startle is disrupted by lesions of the nucleus accumbens. Neuroreport **7**, 1442–1446 (1996). 8856694 10.1097/00001756-199605310-00024

[71] U. Hasson, Y. Nir, I. Levy, G. Fuhrmann, R. Malach, Intersubject synchronization of cortical activity during natural vision. Science **303**, 1634–1640 (2004). 15016991 10.1126/science.1089506

[72] R. Hyon , Similarity in functional brain connectivity at rest predicts interpersonal closeness in the social network of an entire village. Proc. Natl. Acad. Sci. U.S.A. **117**, 33149–33160 (2020). 33318188 10.1073/pnas.2013606117 PMC7777022

[73] R. B. Mars , Comparing brains by matching connectivity profiles. Neurosci. Biobehav. Rev. **60**, 90–97 (2016). 26627865 10.1016/j.neubiorev.2015.10.008 PMC6485474

[74] W. Liu, N. Kohn, G. Fernández, Intersubject similarity of personality is associated with intersubject similarity of brain connectivity patterns. Neuroimage **186**, 56–69 (2019). 30389630 10.1016/j.neuroimage.2018.10.062

[75] P. Hall, S. R. Wilson, Two guidelines for bootstrap hypothesis testing. Biometrics **47**, 757–762 (1991).

[76] G. Chen , Untangling the relatedness among correlations, part I: Nonparametric approaches to inter-subject correlation analysis at the group level. Neuroimage **142**, 248–259 (2016). 27195792 10.1016/j.neuroimage.2016.05.023 PMC5114176

[77] N. Kriegeskorte, M. Mur, P. Bandettini, Representational similarity analysis—connecting the branches of systems neuroscience. Front. Syst. Neurosci. **2**, 4 (2008). 19104670 10.3389/neuro.06.004.2008 PMC2605405

