# Supplementary material for: Individuals who see the good in the bad engage distinctive default network coordination during post-encoding rest
Source: Proc Natl Acad Sci U S A. 2023 Dec 27;121(1):e2306295121. doi: 10.1073/pnas.2306295121 (PMC10769837; doi:10.1073/pnas.2306295121)
Supplement: Supplementary file 1 — Appendix 01 (PDF) [file pnas.2306295121.sapp.pdf]

Replication of Primary Results with a Different Parcellation Scheme

We performed follow-up analyses to help ensure the observed results were not specific to our chosen parcellation scheme. As reported in the primary manuscript, we wanted to objectively define default network brain regions while also ensuring that the regions selected are functionally relevant to psychological constructs. For this reason, in the primary manuscript, we used the k=50 whole brain parcellation that used k-means clustering to isolate meta-analytic coactivations from Neurosynth (1,2).

Here, we report results from the mindboggle parcellation (3), which includes a manually labeled parcellation atlas, following the labeling protocol of Desikan et al. (4). This parcellation was chosen because it also separates, in relatively large swaths, the medial prefrontal cortex into ventral and dorsal regions. Using the mindboggle parcellation, we again observed that the Anna Karenina model testing whether idiosyncratic functional connectivity profiles between default network regions predicted more positive patient memories was significant (r = .270, p = .016, permutation test).

In contrast, the same analysis for the baseline rest phase, post-science rest phase, patient video watching, and science video watching were all non-significant (Supplementary Table 1). Additionally, partial mantel tests again demonstrated that the Anna Karenina model testing whether idiosyncratic functional connectivity profiles between default network regions predicted more positive patient memories remained significant when controlling for connectivity during the other experimental phases (Supplementary Table 2).

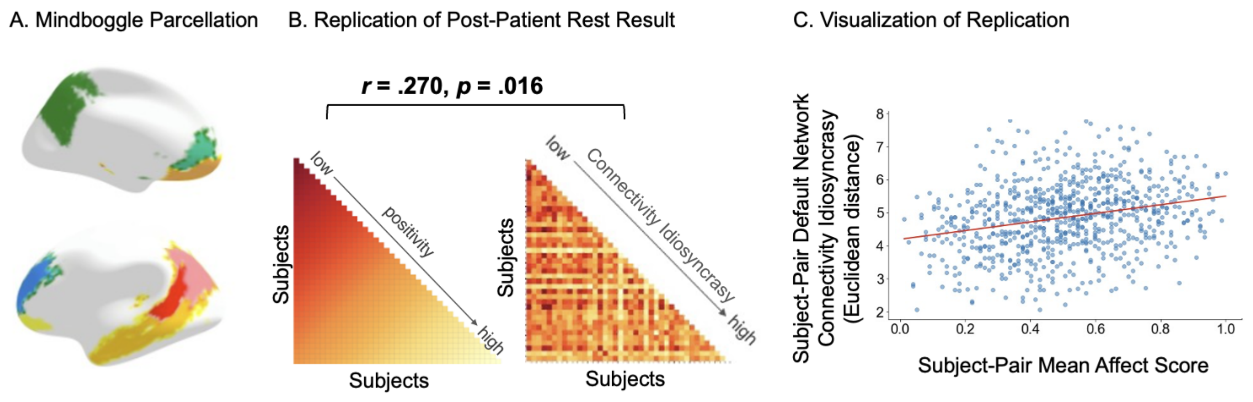

Supplementary Figure 1. Panel A shows the default network ROIs from the mindboggle parcellation scheme. Panel B shows that the Anna Karenina model testing whether participants with more idiosyncratic functional connectivity profiles have more positive memories is significant. Panel C visualizes this result.

	Baseline Rest	Post-Science Rest	Patient Video Watching	Science Video Watching
Anna Karenina Model	*r* = -.064<br>*p* = .559	*r* = .115<br>*p* =.328	*r* = -.162<br>*p* = .149	*r* = -.083<br>*p* = .413

Supplementary Table 1. Results from the Anna Karenina model with the mindboggle parcellation tested on each phase of the experiment that was not post-patient rest. P-values derived from permutation tests of significance.

	Controlling for Baseline Rest	Controlling for Post-Science Rest	Controlling for Patient Video Watching	Controlling for Science Video Watching
Anna Karenina Model for Post-Patient Rest, Controlling for Other Phases	*r* = .287<br>*p* =.009	*r* = .299<br>*p* =.007	*r* = .317<br>*p* = .004	*r* = .245<br>*p* = .030

Supplementary Table 2. Results from the partial mantel tests using the mindboggle parcellation. The partial mantel tests assess whether the observed relationship between idiosyncratic default network functional connectivity during post-patient predicted more positive patient memories, while controlling for connectivity during the other experimental phases. P-values derived from permutation tests of significance.

References

1. de la Vega, A. Chang, L.J. Banich, M.T. Wager, T.D. Yarkoni, T. Large-Scale Meta-Analysis of Human Medial Frontal Cortex Reveals Tripartite Functional Organization. J. Neurosci. 36, 6553–6562 (2016).
2. Yarkoni, T. Poldrack, R.A. Nichols, T.E. Van Essen, D.C. Wager, T.D. Large-scale automated synthesis of human functional neuroimaging data. Nat. Methods. 8, 665–670 (2011).
3. Klein, A. & Tourville, J. 101 labeled brain images and a consistent human cortical labeling protocol. Frontiers in neuroscience, 6, 171 (2012).
4. Desikan, R. S. Ségonne, F. Fischl, B. Quinn, B. T. Dickerson, B. C., Blacker, D. ... & Killiany, R. J. An automated labeling system for subdividing the human cerebral cortex on MRI scans into gyral based regions of interest. Neuroimage, 31, 968-980 (2006).
